# Spices in a Product Affect Emotions: A Study with an Extruded Snack Product †

**DOI:** 10.3390/foods6080070

**Published:** 2017-08-18

**Authors:** Brandon Bell, Koushik Adhikari, Edgar Chambers IV, Sajid Alavi, Silvia King, Mark Haub

**Affiliations:** 1 WhiteWave Foods Company, Broomfield, CO 80021, USA; chipperwayne@gmail.com; 2 Food Science & Technology, University of Georgia, Griffin, GA 30223, USA; 3 Center for Sensory Analysis and Consumer Behavior, Kansas State University, Manhattan, KS 66502, USA; eciv@ksu.edu; 4 Grain Science and Industry, Kansas State University, Manhattan, KS 66506, USA; salavi@ksu.edu; 5 Silvia C. King Consulting LLC, Maineville, OH 45039, USA; sc@silviacking.com; 6 Food, Nutrition, Dietetics and Health, Kansas State University, Manhattan, KS 66506, USA; haub@ksu.edu

**Keywords:** emotion, spices, extruded snack, overall liking, clinical, antioxidants

## Abstract

Food commonly is associated with emotion. The study was designed to determine if a spice blend (cinnamon, ginger, nutmeg, and cloves) high in antioxidants can evoke changes in consumer emotions. This was an exploratory study to determine the effects of these four spices on emotions. Three extruded, dry snack products containing 0, 4, or a 5% spice blend were tested. One day of hedonic and just-about-right evaluations (*n* = 100), followed by three days of emotion testing were conducted. A human clinical trial (*n* = 10), using the control and the 4% samples, measured total antioxidant capacity and blood glucose levels. The emotion “Satisfied” increased significantly in the 5% blend, showing an effect of a higher spice content. The 4% blend was significantly higher in total antioxidant capacity than the baseline, but blood glucose levels were not significantly different.

## 1. Introduction

### 1.1. Food and Emotion

The use of emotions to evaluate food is a reasonably new consumer research tool [1]. Considerable work is available on the impact of emotion on eating [2], and on the impact of odor on emotion [3,4]. For example, Rétiveau et al. [5], and Freyberg & Ahren [6] found significant relationships between odor perception of fragrances and mood or emotion in female participants, and Kergoat et al. [7] found that the emotional construct “affect” had an effect on the perception of tactile softness. Similarly, the impact of emotion on eating [8,9] also has been studied and the alteration of moods can consciously or subconsciously affect the liking of a particular food [10]. Moskowitz et al. [11] showed that the promise of emotional benefits led to higher utility scores for acceptance in a conjoint study in a snack food category.

However, little work on the impact of food on emotion is available, especially as it might relate to helping product developers understand responses to products [12]. Steptoe et al. [13] found that food affects the way we feel, and that mood is a key determinant of food choice, and Gibson [14] suggested that one’s mood or emotion could be affected by our food choice and vice versa. Kuenzel et al. [15] found that consumers could be conditioned to associate novel flavors, but not familiar flavors, with various emotions, and that subsequently could impact liking scores. However, no research was found on the impact of specific active ingredients (e.g., nutraceuticals such as spices) in actual food products on emotion. 

Consumers often eat foods, consciously or subconsciously, that coincide with their current mood status or the mood outcomes desired. Hedonics, food, mood, and emotions often are interrelated because of the food’s effect on the body. When people are hungry, they are often irritable. The consumption of foods therefore will increase satiation, which in turn induces calming and positive thoughts. Thus, eating generally is a positive experience in healthy consumers [16]. 

Data are lacking concerning the link between acceptance and emotional intensities. Some central location tests (CLTs) have shown a positive correlation between overall liking and positive emotional intensities, while other tests have shown little to no correlation [12]. Those authors suggested that such findings might provide a reason why a product can be well liked in consumer tests, but fail in the marketplace, as emotions may be playing a role in the buying decision. Part of the issue may be the population studied. Piqueras-Fiszman et al. [17] showed differences in younger and older consumers in terms of the emotional impact of food labels. In order to study the effect of food on emotion, King and Meiselman [12] published an emotion questionnaire that targets information for use in the commercial food context. The EsSense Profile™ was designed to differentiate among completely different food products, as well as among similar foods with slight differences. Cardello et al. [18] measured emotional responses to foods (actual tasting) and compared the responses elicited by food names (no tasting) using the EsSense Profile™. They found that both methods evoked a range of emotions, and the correlation coefficients varied between +0.66 and +0.83, depending on the food item. Principal components analysis also revealed a similar pattern, where both the food name and actual testing were positively related.

Spices are widely used around the world, and many aromatic compounds in spices have been found to impact emotion. Chrea et al. [3] showed that various odors, such as spices could affect emotion. 

### 1.2. Spices and Antioxidant Activity

Antioxidants are molecules that slow or prevent the oxidation of other molecules that contain substrates susceptible to oxidation [19]. Spices such as cinnamon, ginger, nutmeg, and cloves contain powerful antioxidants.

Cinnamon is one of the most commonly used spices worldwide, and its health benefits have been identified in animal, though not many human studies [20]. Studies have shown that cinnamon decreases blood glucose and insulin levels in diabetic rats, and rats fed high sugar diets [21,22]. Research is scarce as to the bioavailability of antioxidants from spices in human clinical trials [23]. In cell culture studies, ginger was shown to scavenge free radicals, as well as inhibit peroxidation because of its antioxidant activities [24,25]. Nutmeg contains powerful antioxidants equivalent to approximately 2000 ORAC/tsp (oxygen radical absorbance capacity per teaspoon). In vitro, the phytochemicals in nutmeg have demonstrated strong bioactivity; the antioxidants scatter free radicals at a moderate rate [25]. Cloves have the highest amount of antioxidants (125.5 mmol/100 g) when plant sources are considered. In vitro, cloves have demonstrated the strongest radical scavenging activity [26].

We found little research on the effect of spices on mood and emotion, or human clinical studies outlining the benefits of foods with functional spice blends. The major objective of this study was to determine the effects of a spice blend (cinnamon, ginger, nutmeg, and cloves) on the emotions of consumers. A second objective was to study the differences between emotions of consumers who evaluated products one time, and consumers who evaluated the products repeatedly. A third objective was to compare the health implications (blood glucose effect and bioactivity of antioxidants) of the spices in a small clinical trial to determine if benefits that might be determined in sensory studies were based on the physico-chemical responses in blood. This was an exploratory study to find out the impact that spices might have on emotions.

## 2. Materials and Methods 

### 2.1. Preparation of the Extruded Samples Containing the Spice Blend

A spice blend was created using ground cinnamon (57%), ground dried ginger (22%), ground nutmeg (15%), and ground cloves (6%) by McCormick and Company (experimental samples; Hunt Valley, MD, USA). The composition of the spice blend used in the study was given to the first author by McCormick’s Research & Development team. An apple-based extruded snack containing 0 (control), 4, and 5 g/100 g of the spice blend (final composition) was used as a carrier food for this study. The snack products were made with corn flour (Bunge Milling Inc., St. Louis, MO, USA), apple pomace (Tree Top Inc., Selah, WA, USA), and table salt (1%). The products were extruded in a Micro-18 twin-screw extruder (American Leistritz, Somerville, NJ, USA) under controlled processing conditions. The extruder had a feed rate of approximately 2.5 kg/h, and a screw speed of 350 rpm. The temperature profile from the inlet to the outlet was: 50, 65, 80, 90, 110, and 120 °C, respectively. The conditions for extrusion were determined by pre-trials at the extrusion lab. 

The composition of the snack products (dry-weight basis) is given in Table 1. The “control” throughout the paper is the non-spiced sample, and the two test samples are referred to as the “4% blend” and the “5% blend”, identifying their respective spice blend proportions. A sugar coating (28%), using simple syrup (1:1 ratio), was sprayed on the outside of the product. The products were stored in food-grade plastic bins with lids (36 L; Sterilite Corporation, Townsend, MA, USA) at room temperature (22 ± 1 °C) until packaged in individual sandwich-sized Zip-Loc^®^ bags (S.C. Johnson & Son, Inc., Racine, WI, USA) for the various evaluations. 

The spices and Oxygen Radical Absorbance Capacity (ORAC) values were: cinnamon (267,536 µmol Trolox Equivalent or TE/100 g), ginger (28,811 µmol TE/100 g), nutmeg (39,800 µmol TE/100 g), and cloves (314,446 µmol TE/100 g) [27]. 

### 2.2. Consumer Studies

#### 2.2.1. Consumers 

One hundred consumers (61 females and 39 males) were recruited from Manhattan, Kansas and surrounding areas to participate in four consumer tests. The participants were recruited on the basis that they could attend all four sessions, had no known food allergies, regularly consumed cinnamon, cloves, and nutmeg; ate snacks, and were between 18 and 69 years old. Age ranges were 18–24 (19%), 25–40 (42%), 41–55 (28%), and 56–69 (11%). A total of four consumer tests were conducted during a two-week period. A 9-point hedonic scale, anchored from “Dislike Extremely” to “Like Extremely”, was used to collect the data. Prior to all testing (hedonic, emotion, and clinical), participants signed an informed consent, approved by the Institutional Review Board (IRB) of Kansas State University, which outlined the potential risks, the methods, and the purpose of the research.

#### 2.2.2. Samples and Testing 

Approximately 10 g of each of the three products was served using a sequential monadic test design. The products were labeled with a randomly generated 3-digit code and served in sandwich-sized Ziploc bags (S.C. Johnson & Son, Inc., Racine, WI, USA). The consumers completed a questionnaire with liking (overall, texture, appearance, and flavor). The serving order was randomized and balanced.

### 2.3. Consumer Studies—Emotion Testing

The final three sessions were used to evaluate consumers emotions associated with each product, and determine what effects the spices had on consumers’ emotions. Each consumer tested one sample per session. The same 100 consumers were used from the hedonic test. The emotion ballot listed 39 emotions (EsSense Profile™) and was completed using a 5-point scale [12], with each particular emotion scored from “Not at All” (1) to “Extremely” (5). Consumers were asked to fast for at least two hours prior to the emotion evaluation. Twenty-five consumers evaluated the control in all the three sessions; 25 evaluated the 4% blend in all the three sessions; 25 evaluated the 5% blend in all the three sessions; and, 25 evaluated all three products, one on each day. Each consumer filled out three emotion questionnaires: one prior to consumption, one immediately after consumption of one serving (30 g) of product, and the third questionnaire 1 h after consumption. Overall liking data were collected immediately after consumption of the product during the second emotion evaluation. Each eating session lasted approximately 25 min. The consumers completed the last emotion ballot an hour later, away from the test location, and were asked not to eat during this one-hour period of time. All ballots were to be returned in person or by mail.

Post0-∆ scores were calculated by subtracting individual immediate-post emotion scores from pre-consumption emotion scores. The post0-∆ scores for each blend were the average of the 25 consumers who ate the product three times, along with the 25 consumers who ate the product once (for a total of 100 non-independent evaluations). The post1h-∆ scores were derived the same way. The 1-h post-consumption emotion scores were subtracted from the pre-consumption emotion scores to obtain the value. The delta scores were calculated in order to offset the day-to-day variations in consumers’ emotions, and standardize the initial emotion intensities of all of the consumers. 

### 2.4. Clinical Study

#### 2.4.1. Subjects and Samples 

Ten subjects, a typical number for a pilot clinical trial, were recruited for the clinical portion of the research. Participants were recruited on the basis of having no known food allergies, no aversion to cinnamon, and willingness to have blood drawn. The participants consumed two servings of the product (60 g), and gave four blood samples over a 2-h period. Participation was voluntary and each had to fast for at least 10 h prior to the test. The participants came in a total of four times over a four-week period. 

The 4% spice blend sample was selected for the clinical study to be assessed against the control because the Just-About-Right (JAR) attributes (data not presented) were optimized at this level. There are currently no established recommended daily allowances (RDA) for antioxidants; however, the range of 3000 to 5000 (ORAC units) has been recommended by health experts [28]. The ORAC value of the 4% blend was a 2204 µmol TE/serving. Baseline measurements were taken to have a standard against which to compare.

#### 2.4.2. Total Antioxidant Capacity 

Venous samples were taken prior to consumption (0 min/baseline), and at 120 min post-consumption. A trained phlebotomist took approximately 2.5 mL of blood intravenously using a 5 mL disposable syringe (Nipro Medical Corp., Miami, FL, USA), and an eclipse needle (0.6 mm × 25 mm, BD, Franklin Lakes, NJ, USA). The blood was transferred immediately to 6 mL vacutainers (BD K2 EDTA 10.8 mg, Franklin Lakes, NJ, USA) to prevent blood coagulation. The vacutainers were centrifuged (CRU-5000 centrifuge, Damon, IEC division, Nutley, NJ, USA) at 4 °C for 10 min to separate the plasma from the blood. The plasma was then transferred via transfer pipet to a 1.7 mL microcentrifuge tube and placed in a freezer at −20 °C. Total antioxidant capacity (TAC) Colorimetric Assay Kit (Catalog #K274-100, BioVision Research Products, Mountain View, CA, USA) was used to determine the bioavailability of the antioxidants in the samples, as outlined in their datasheet. For the analysis, 3.0 µL of thawed plasma was assayed using TAC assay kits. The total antioxidant capacity was calculated using the following formula:

(1) $$\mathrm{Total}\;\mathrm{Antioxidant}\;\mathrm{Capacity}=\frac{(\mathrm{Sample}\;\mathrm{Absorbance}-\mathrm{Blank}\;\mathrm{Absorbance})-\mathrm{Intercept}}{\mathrm{Slope}\;\mathrm{of}\;\mathrm{Standard}\;\mathrm{Curve}\times \mu L\;\mathrm{of}\;\mathrm{Sample}}$$

The results of Total Antioxidant Capacity were calculated as nmol/µL of plasma or mM Trolox Equivalent (TE).

#### 2.4.3. Glycemic Index

For glycemic index measurements [29], finger prick blood samples were taken via high flow safety lancets (1.8 mm depth, Fisher Scientific, Houston, TX, USA) at 30 and 60 min post-consumption. Baseline (0 min) and 120 min time points were obtained from the venous blood samples. The glycemic index measurements were analyzed with an automated glucose analyzer (YSI 2300 STAT PLUS, Rankin Biomedical Corp., Clarkston, MI, USA) immediately after blood collection to prevent blood coagulation. The blood glucose was reported as mmol/L. The Glycemic Index was calculated as the incremental area under the curve using the trapezoidal method (GraphPad Prism version 5.1; GraphPad Software, La Jolla, CA, USA) from the blood glucose data.

### 2.5. Data Analysis

Acceptability data were analyzed by analysis of variance (ANOVA) using PROC GLM in SAS^®^ version 9.2 (SAS Institute Inc., Cary, NC, USA). Post-hoc mean separation used Fisher’s Least Significant Difference (LSD). All significant differences were determined at the 95% confidence level (α = 0.05).

The mean scores and difference from the baseline emotion scores (difference = emotion scores immediately after consumption or emotion scores 1 h after consumption, minus emotion scores before consuming a product) for each product were calculated using Microsoft Excel^®^ 2007 and presented as radar plots. 

The Total Antioxidant Capacity measurements and Glycemic Index data (area under the curve data) were subjected to a one-way ANOVA, and means were separated using Fisher’s LSD at α = 0.05. 

## 3. Results and Discussion

### 3.1. Consumer Studies

#### 3.1.1. Overall Liking Data 

Overall liking, appearance liking, flavor liking, and texture liking were assessed by all 100 consumers, and showed significant differences (*p* < 0.05) based on the blend of the snack products. Table 2 shows that the hedonic differences between samples are minimal with all of the lab produced samples scoring in the neutral range. The appearance of all three products was disliked slightly, and this might have influenced the other attributes as well. The 4% blend was not liked for its texture, as compared to the 5% spice blend.

**Figure 1:**
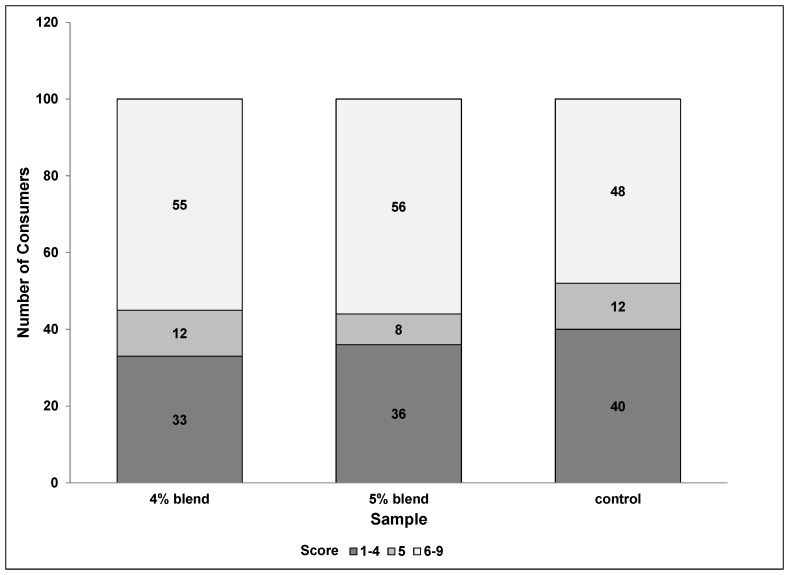
Overall liking score groupings based on frequency of response.

In Figure 1, the overall liking scores have been grouped for each of the three samples. These data are a good supplement to the mean averages because it shows if there are any outliers that are skewing the results. Over half of the consumers, 56% and 55%, respectively, rated the overall liking at least a “6” (at least liked slightly) for the 5% blend and 4% blend, whereas only 48% of consumers rated the control that high. This suggests that the spice blend increased overall liking for the average consumer, increasing overall acceptability.

#### 3.1.2. Overall Liking—Hedonic vs. Emotion Data

The average overall liking scores on the emotion ballot either stayed constant or increased from the hedonic ballot average scores. The only difference between the two tests was the amount of sample consumed (10 g for the hedonic test and 30 g for the emotion test). It appears either the product became more acceptable after eating a larger amount, or that the overall liking was increased because of better product familiarity after repeated exposure. The averages of overall liking for Days 1, 2, and 3 were within a similar range for all three samples, confirming that the consumers may have used the hedonic ballot overall liking as a “warm-up” for such a novel product, and the scores became more consistent as the consumers gained product familiarity.

### 3.2. Emotion Differences by Blend and Time 

Post-hoc mean separation using Fisher’s LSD identified eight significant constructs for both 4% and 5% spice blend products, while seven constructs were significant for the control. Interestingly, “Calm” was the only emotion that was significantly different in all three samples. Calmness significantly decreased over time from immediate post-consumption to 1-h post-consumption across all three blends. This finding is contrary to the research conducted by Macht et al. [10], who found that consumption of food reduces irritability and increases calmness because of the increase of satiation. However, in our study, consumers recorded their initial mood score in a controlled setting, whereas the later scores were given in whatever setting they were in one-hour later, often a busy work environment. That could explain why calmness was higher before the snack intake than one hour later.

The emotion “Satisfied” also is of particular interest because it only significantly differed in the 5% blend. Satisfaction increased from both pre to post0 (Post0-∆), and post0 to post1h (Post1h-∆) which might have been because of the higher spice level because the 4% blend did not show a similar trend. Perhaps a spice level of 5% blend for this blend was the threshold to have an impact on this emotion.

**Figure 2:**
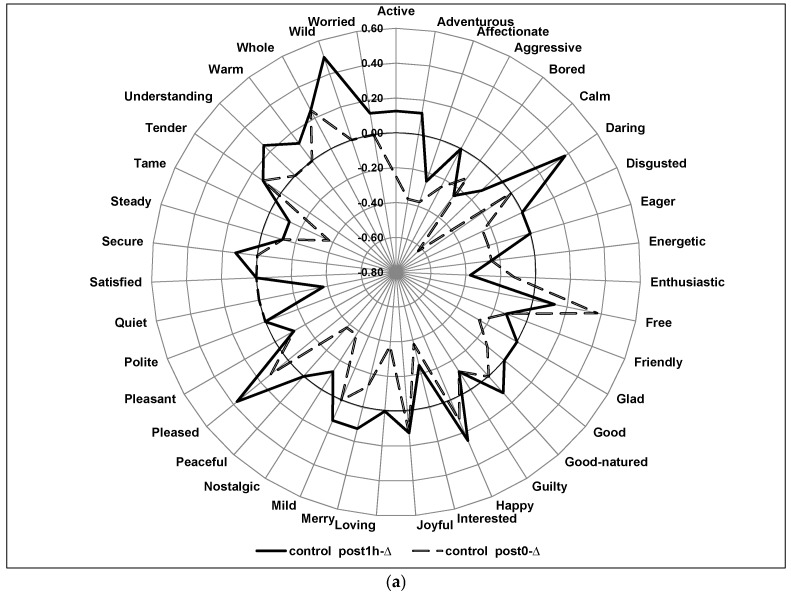

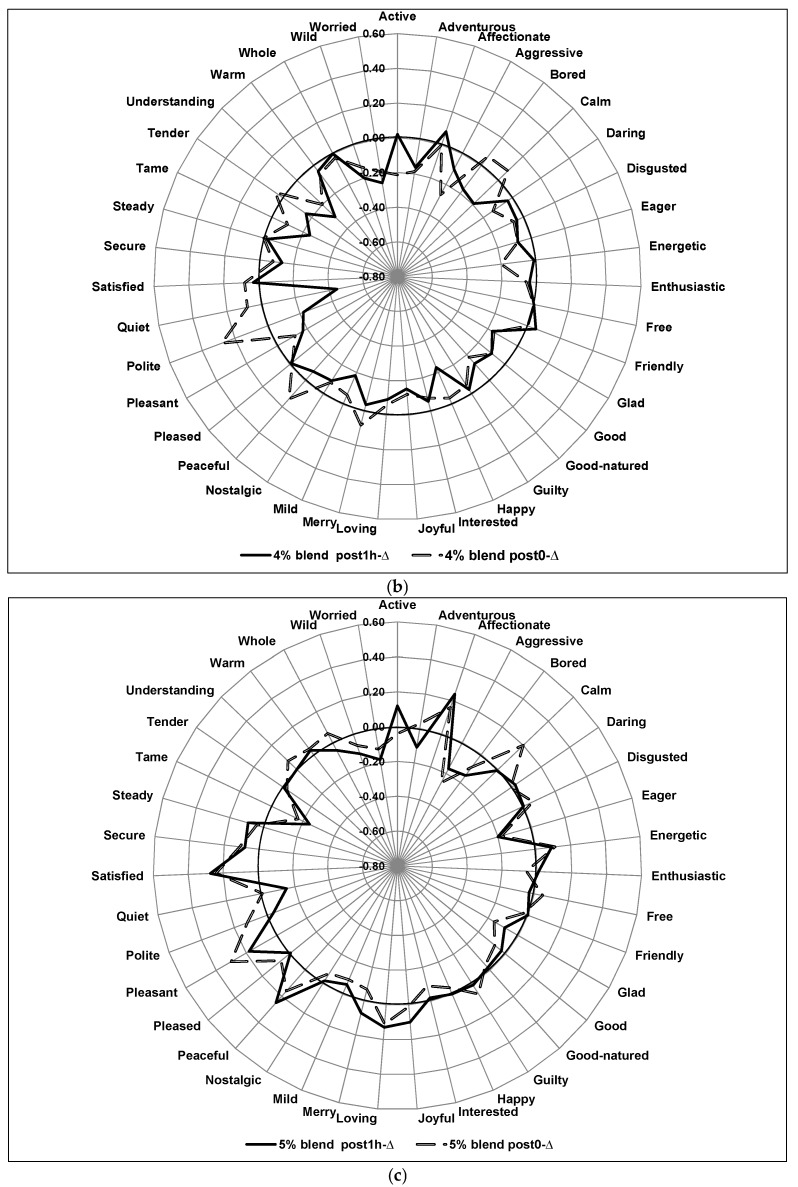
(**a**) Emotion intensity changes over time for each individual product: Control. (**b**) Emotion intensity changes over time for each individual product: 4% Blend. (**c**) Emotion intensity changes over time for each individual product: 5% Blend. Legend—Post0-∆ = immediate-post consumption—pre consumption; Post1h-∆ = one-hour post consumption—pre consumption.

Figure 2 highlights the differences between samples for emotions over time (post0-∆ scores vs. post1h-∆ scores). For the control (Figure 2a), the post1h-∆ scores were slightly higher than post0-∆ scores, in general. In most cases, the pre-consumption emotion scores were more intense than the post and post1h emotion scores as many of the peaks and valleys were either near or below 0.00. The post1h-∆ scores had large positive peaks for the terms, ‘Wild,’ ‘Daring’, and ‘Adventurous’, while the post0-∆ score had a large positive peak for the emotion ‘Free.’

Figure 2b,c shows the post0-∆ scores vs. post1h-∆ scores for the 4% blend and 5% blend, respectively. For the 4% and 5% blends, the post0-∆ scores are slightly higher than post1h-∆ scores, in general. This is opposite to the trend for the control sample as the post1h-∆ scores were slightly higher than post0-∆ scores for that sample. Also noteworthy is the observation that the range of the delta scores is narrower for the 4% and 5% blends, in general, when compared to the control. For the 4% blend, the emotion terms that stand out are: ‘Calm’, ‘Bored’, ‘Peaceful’, Polite’, and ‘Quiet’. For the 5% blend, ‘Satisfied’, ‘Pleasant’, ‘Peaceful’, and ‘Affectionate’ stood out as both the post0-∆ scores and post1h-∆ scores had large, positive peaks. The significance of the differences in the intensity of these emotions in this particular context deserves future research in order to gain a better understanding of their particular meaning. The terms may have a significant impact on product acceptance or prove a way to more fully understand a particular product. 

From a physiological standpoint, it appears that the spices in our snack products may have a suppressing effect on these certain emotions. This is similar to information found by Rétiveau et al. [5] who found that certain emotions were lessened in subjects wearing equally pleasant fragrances for up to 3 h or as long as the perfumes were still discernable. As with that study, the data from our study, where liking was similar across the products, suggests that the differences in emotion are not dependent on differences in acceptance.

**Figure 3:**
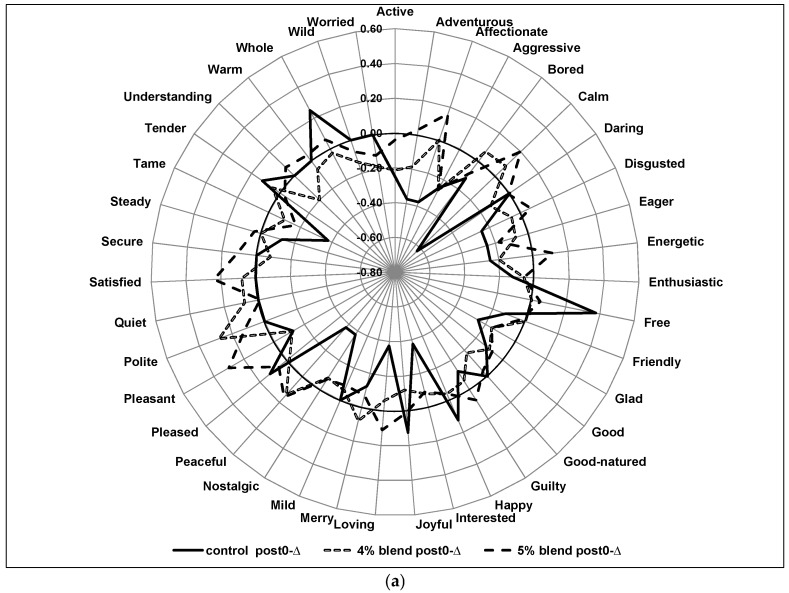

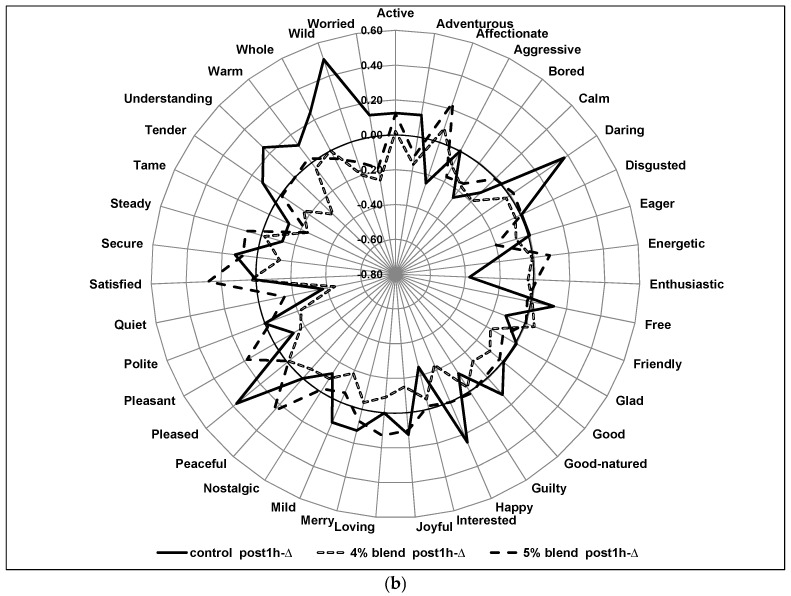
(**a**) Emotion intensity changes over time for all the products: Post0-∆ scores. (**b**) Emotion intensity changes over time for all the products: Post1h-∆ scores. Legend—Post0-∆ = immediate-post consumption—pre consumption; Post1h-∆ = one-hour post consumption—pre consumption.

Figure 3 differentiates the samples from one another at the post0-∆ and post1h-∆ time points. With a few exceptions, the 5% blend post0-∆ scores seem slightly higher than the 4% blend and control post0-∆ scores (Figure 3a). Another notable trend is that the 4% and 5% blends seem relatively stable, both the ∆ scores usually ranging from 0.2 to −0.2, whereas the control had more variance, as seen by the plethora of peaks and valleys and wider range. Figure 3b shows the variation in the intensities of emotion for the post1h-∆ scores for the three different samples. Again, the 4% and 5% blends averages were more stable than the control. But, unlike the trend seen for the post0-∆ scores, the control post1h-∆ scores appear slightly higher than the scores from spiced blends, on average. The scores for control showed larger positive peaks that are much higher than the other two blends for the emotions ‘Understanding’, ‘Wild’, ‘Worried’, ‘Daring’, ‘Happy’, and ‘Pleased’. The particular significance or underlying meaning of these terms needs to be further examined, bridging the gap between product development and psychology. If these emotions positively affect product acceptance, than the developed samples would require reformulation to increase these emotions. Lastly, the 5% blend is higher in the ‘Satisfied’ intensity when compared to the 4% blend and the control sample. This suggests the possibility that the extra 1% spice concentration in the 5% blend sample elicits a stronger feeling of ‘satisfaction’ with consumers. 

Benton & Donohoe [30] identified that sweet, carbohydrate-rich foods and some micronutrients increase positive emotions. Endorphins, which are neurotransmitters in the brain that relieve pain and produce a feeling of wellness and satisfaction, are thought to be responsible for this positive affect. Perhaps, the sweetness of the extruded products was not high enough to evoke such an endorphin response. The control sample had little to no change in ‘Satisfaction’ intensity, as evidenced in Figure 3a,b. However, the phytochemicals or other constituents in the spice blends may have caused the increase in ‘Satisfaction’, as shown in the spice blend samples. It would be interesting to further increase the spice concentration in the blend to gain a better understanding of the interaction between the spices and consumer satisfaction. The composition of the spice blends could also be altered in an attempt to maximize consumer satisfaction. 

With a better understanding of how emotions relate to product success, product developers would have another tool to measure potential consumer acceptance. This research could prove beneficial because, if certain emotions were confirmed as predictors of potential market success, then product developers could reduce their risks of introducing a product that is prone to fail, as well as reduce the lengthy testing time that extended use tests require.

### 3.3. Clinical Study

#### 3.3.1. Antioxidant Levels in the Plasma 

The Total Antioxidant Capacity (TAC) results are shown in Table 3. The plasma TAC values for both control and 4% spice blend increased slightly after 2 h. The control sample values, at 0 and 120 min, were slightly lower than the 4% spice blend. Both these comparisons were not significant (*p* > 0.05).

#### 3.3.2. Glycemic Index 

**Figure 4:**
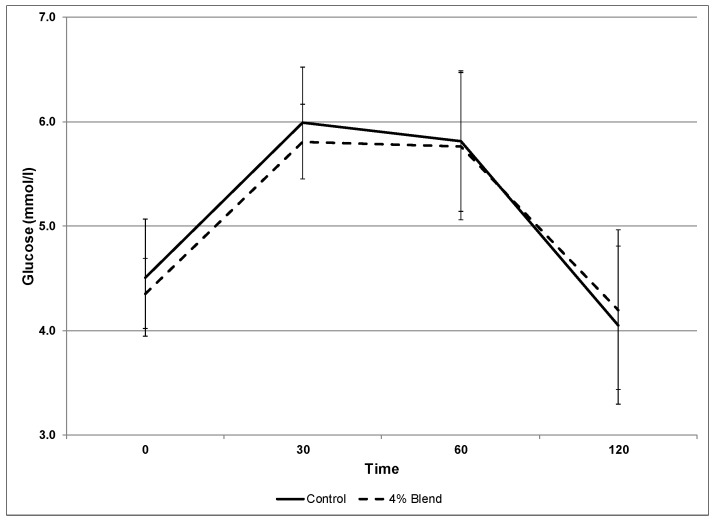
Comparison of blood glucose value averages (*n* = 10) at 0, 30, 60, and 120 min for the control and 4% blend.

Cinnamon made up 57% of the spice blend, comprising just over 2% of the total blend. The 4% spice sample, and the control showed no significant differences (Table 3). The curves comparing the average glucose values for the control, and the 4% blend are nearly identical (Figure 4). The finding that cinnamon and the other spices have no effect on lowering blood glucose is reflective of other diabetic studies [31,32]. The finding also suggests that the alteration of emotion was not directly related to the glycemic index, a measure of “sugars” in the blood.

## 4. Conclusions

The additions of the spice blends to the control base did not significantly affect overall liking, flavor liking, or appearance liking. Average appearance liking for all samples was low, possibly influencing the scores of the other attributes. Just-about-right scores appeared to favor the 4% blend sample over the 5% blend and control samples, and hence was used for clinical portion of the study.

The spice blends had an effect on the intensity of emotions that appeared independent of acceptance or the clinical glycemic index. The duration of a particular emotion’s intensity was also affected by spice or lack of spice. Spices seem to increase certain emotional intensities immediately after consumption, but these intensities generally diminished after one hour post-consumption. The emotion “Satisfied” was enhanced by the 5% spice blend. Inconsistencies in the data suggest future research is needed to better understand the implications of how specific emotions affect consumer acceptability. The main limitation of the study was the sample size for both the emotion and the clinical study. Although each sample including the control in the emotion study was replicated three times with the same group of 25 consumers to understand the effects of the spice blends on emotions, a larger size, preferably ≥50, may be needed to understand the effects of the spices on emotion. An extended clinical study might be needed to determine the effects of the spice blends on the glycemic index.

## Figures and Tables

**Table 1:** Composition of the extruded snack * (g/100 g of product on dry-weight basis).

Ingredients	Control	4% Blend	5% Blend
Total Spice Blend	N/A	4.0	5.0
Salt	1.0	1.0	1.0
Corn Flour	77.0	73.9	73.1
Apple Fiber	22.0	21.1	20.9
Moisture of the final product (% *w*/*w*)	8.8	9.1	9.8

* 14% sucrose coating was applied after extrusion.

**Table 2:** Mean hedonic scores for the extruded snacks.

Sample	Overall Liking	Appearance Liking	Flavor Liking	Texture Liking
5% Blend	5.4 ^a^	4.0 ^a^	5.3 ^a^	5.6 ^a^
4% Blend	5.4 ^a^	3.9 ^a^	5.3 ^a^	5.2 ^b^
Control	5.2 ^a^	4.0 ^a^	5.2 ^a^	5.6 ^a^

^a,b^ Column means with the same letter are not significantly different (*p* > 0.05).

**Table 3:** Results from the clinical study (*n* = 10).

**Total Antioxidant Capacity (Plasma)**	**mM TE ± SD**
Control—antioxidant activity at 0 h	19.4 ± 1.8 ^c^
4% Spice Blend—antioxidant activity at 0 h	20.3 ± 1.1 ^a,b^
Control—antioxidant activity after 2 h	20.0 ± 1.7 ^b,c^
4% Spice Blend—antioxidant activity after 2 h	20.8 ± 1.8 ^a^
**Glycemic Index**	**Average Area Under the Curve ± SD**
Control	117.4 ± 47.0 ^a^
4% Spice Sample	115.6 ± 47.0 ^a^

^a,b,c^ Column means (Total Antioxidant Capacity and Glycemic Index) with the same letter are not significantly different (*p* > 0.05).

## References

[1] Kenney E., Adhikari K. (2016). Recent developments in identifying and quantifying emotions during food consumption. J. Sci. Food Agric..

[2] Macht M. (2008). How emotions affect eating: A five-way model. Appetite.

[3] Chrea C., Grandjean D., Delplanque S., Cayeux I., Le Calvee B., Aymard L., Velazco M., Sander D., Scherer K.R. (2009). Mapping the semantic space for the subjective experience of emotional responses to odors. Chem. Senses.

[4] Seubert J., Rea A.F., Loughead J., Habel U. (2009). Mood induction with olfactory stimuli reveals differential affective responses in males and females. Chem. Senses.

[5] Rétiveau A., Chambers IV E., Milliken G. (2004). Common and specific effects of fine fragrances on the mood of women. J. Sens. Stud..

[6] Freyberg R., Ahren M.-P. (2011). A preliminary trial exploring perfume preferences in adolescent girls. J. Sens. Stud..

[7] Kergoat M., Giboreau A., Nicod H., Faye P., Diaz E., Beetschen M.-A., Meyer T. (2012). Consumer preference for tactile softness: A question of affect intensity?. J. Sens. Stud..

[8] Rollins B.Y., Riggs N.R., Spruijt-Metz D., McClain A.D., Chou C-P., Pentz M.A. (2011). Psychometrics of the eating in Emotional Situations Questionnaire (EESQ) among low-income Latino elementary-school children. Eat. Behav..

[9] Polivy J., Herman C.P., Deo R. (2010). Getting a bigger slice of the pie. Effects on eating and emotion in restrained and unrestrained eaters. Appetite.

[10] Macht M., Haupt C., Salewsky A. (2004). Emotions and eating in everyday life: Application of the experience-sampling method. Ecol. Food Nutr..

[11] Moskowitz H., Silcher M., Beckley J., Minkus-Mckenna D., Mascuch T. (2005). Sensory benefits, emotions and usage patterns for olives: Using internet-based conjoint analysis and segmentation to understand patterns of response. Food Qual. Preference.

[12] King S., Meiselman H. (2010). Development of a method to measure consumer emotions associated with foods. Food Qual. Preference.

[13] Steptoe A., Pollard T.M., Wardle J. (1995). Development of a measure of the motives underlying the selection of food: The food choice questionnaire. Appetite.

[14] Gibson E. (2006). Emotional influences on food choice: Sensory, physiological, and psychological pathways. Physiol. Behav..

[15] Kuenzel J., Zandstra E.H., Lion R., Blanchette I., Thomas A., El-Deredy W. (2010). Conditioning unfamiliar and familiar flavours to specific positive emotions. Food Qual. Preference.

[16] Desmet P., Schifferstein H. (2008). Sources of positive and negative emotions in food experience. Appetite.

[17] Piqueras-Fiszman B., Ares G., Varela P. (2011). Semiotics and perception: Do labels convey the same messages to older and younger consumers?. J. Sens. Stud..

[18] Cardello A.V., Meiselman H.L., Schutz H.G., Craig C., Given Z., Lesher L.L., Eicher S. (2012). Measuring emotional responses to foods and food names using questionnaires. Food Qual. Preference.

[19] Valko M., Leibfritz D., Moncol J., Cronin M., Mazur M., Telser J. (2007). Free radicals and antioxidants in normal physiological functions and human disease. Int. J. Biochem. Cell Biol..

[20] Rao P.V., Gan S.H. (2014). Cinnamon: A multifaceted medicinal plant. Evid. Based Complement. Altern. Med..

[21] Kannapan S., Jayaraman T., Rajasekar P., Ravichandran M., Anuradha C. (2006). Cinnamon bark extract improves glucose metabolism and lipid profile in the fructose-fed rat. Singap. Med. J..

[22] Talpur N., Echard B., Ingram C., Bagchi D., Pruess H. (2005). Effects of novel formulation of essential oils on glucose-insulin metabolism in diabetic and hypertensive rats: A pilot study. Diabetes Obes. Metab..

[23] Dugoua J., Seely D., Perri D., Cooley K., Forelli T., Mills E., Koren G. (2007). From type 2 diabetes to antioxidant activity: A systematic review of the safety and efficacy of common and cassia cinnamon bark. Can. J. Physiol. Pharmacol..

[24] Bone K. (1997). Ginger. Br. J. Phytother..

[25] Shan B., Cai Y., Sun M., Corke H. (2005). Antioxidant capacity of 26 spice extracts and characterization of their phenolic constituents. J. Agric. Food Chem..

[26] Shobana S., Naidu A. (2000). Antioxidant activity of selected Indian spices. Prostaglandins Leukot. Essent. Fat. Acids.

[27] Haytowitz D.B., Bhagwat S. USDA Database for the Oxygen Radical Absorbance Capacity (ORAC) of Selected Foods, Release 2. US Department of Agriculture, Agricultural Research Service. 2010. http://www.ars.usda.gov/nutrientdata/orac.

[28] Prior R., Wu X., Gu L., Jacob R., Cao G., Cook R.A. (2007). Plasma antioxidant capacity changes following a meal as a measure of the ability of a food to alter in vivo antioxidant status. J. Am. Coll. Nutr..

[29] Haub M.D., Hubach K.L., Al-tamimi E.K., Ornelas S., Seib P.A. (2010). Different types of resistant starch elicit different glucose responses in humans. J. Nutr. Metab..

[30] Benton E., Donohoe R. (1999). The effects of nutrients on mood. Public Health Nutr..

[31] Suppapitiporn S., Kanpakso N., Suppapitiporn S. (2006). The effect of cinnamon cassia powder in type 2 diabetes mellitus. J. Med. Assoc. Thail..

[32] Vanschoonbeek K., Thomassen B., Senden J., Wodzig W., Van Loon L. (2006). Cinnamon supplementation does not improve glycemic control in postmenopausal type 2 diabetes patients. J. Nutr..

